# Intravenous iron therapy for patients with heart failure: expanding body of evidence

**DOI:** 10.1002/ehf2.12490

**Published:** 2019-07-16

**Authors:** Christopher Adlbrecht

**Affiliations:** ^1^ Department of Cardiology, Vienna North Hospital – Clinic Floridsdorf and the Karl Landsteiner Institute for Cardiovascular and Critical Care Research Vienna Austria

## 1 Anaemia, iron metabolism, and mitochondrial energy metabolism

Anaemia (haemoglobin <12 g/dL in women and <13 g/dL in men) represents a common co‐morbidity in patients with heart failure (HF). The aetiology of anaemia in these patients is often multifactorial. Chronic gastrointestinal blood loss, vitamin deficiency, reduced iron availability from iron stores or impaired intestinal iron resorption caused by chronic inflammation as well as decreased erythropoietin availability or effect, malnutrition, and of course haemodilution amongst others represent potential reasons.^1^, ^2^ As anaemia is predictive for mortality in HF, a direct association was initially suspected. However, solely increasing haemoglobin by erythropoiesis stimulating drugs failed in improving clinical outcome. In contrast to this, intravenous (i.v.) iron repletion in patients with HF and iron deficiency (ID) led to an increase in exercise capacity and reduced hospitalizations. Importantly, the effect of i.v. iron was independent from baseline haemoglobin levels. ID can lead to impairment of the mitochondrial energy metabolism long before it leads to impaired haematopoiesis detectable in peripheral blood/anaemia. Hepcidin, the central regulator of iron homeostasis, is under normal circumstances down‐regulated in ID, anaemia, and/or hypoxia. Lower hepcidin leads to increased availability from iron stores and to a rise in intestinal iron absorption. However, chronic elevation of inflammatory mediators, as present in disease linked to chronic inflammation, as in HF, prompts to hepcidin up‐regulation. This mechanism leads to ID in HF and also explains why oral iron supplements are ineffective in patients with HFrEF.^3^

## 2 Definition and core evidence for intravenous iron in heart failure

In individuals without a chronic inflammatory state, commonly accepted cut‐off values to define ID are ferritin below 30 μg/L and transferrin saturation below 16%. As ferritin represents an acute phase mediator, these cut‐off levels cannot be used in HF, a disease marked by chronic inflammation. In the double blind FAIR‐HF trial, iron deficient patients with symptomatic HF with reduced ejection fraction with and without anaemia (haemoglobin 9–13.5 g/dL) were randomized to receive i.v. ferric carboxymaltose (FCM) or placebo.^4^ For the reason mentioned earlier, FAIR‐HF used the following definition of ID in HF:

•ferritin < 100 μg/L (‘absolute ID’)

•ferritin 100–299 μg/L if the transferrin saturation was <20% (‘functional ID’)

Intravenous iron repletion improved exercise capacity in symptomatic, ambulatory HF patients,^5^ and reduced HF‐related hospitalizations in a meta‐analysis.^6^ In addition to that, i.v. iron therapy also improved renal function in patients with HF.^7^

## 3 Hypersensitivity reactions of intravenous iron

In general, hypersensitivity reactions can occur when i.v. iron is administered. Therefore, basic safety measures and monitoring should be considered. Newer i.v. iron formulations are considered to be much safer than earlier generations of i.v. iron products. For example, in FAIR‐HF, none of the 304 patients in the group randomized to receive FCM experienced a severe anaphylactic reaction. In another analysis of 1000 FCM administrations, no case of anaphylaxis was observed and moderate‐to‐severe hypersensitivity reactions including moderate‐to‐severe hypotension occurred in 0.7% of the administrations.^8^ A helpful practical guidance for the management of patients to be treated with i.v. iron has been recently published taking up‐to‐date evidence into account.^9^

Although several different i.v. iron preparations are available, iron sucrose and FCM were thoroughly tested in HF patients. FCM has the additional benefit of well‐established high dosage administration within a short application time.^5^, ^10^

## 4 Prognostic implications of congestion, cardiac decompensation, and potential impact of iron on congestion

Plasma volume increase is a typical feature of worsening HF, which is thought to be caused by neurohormonal activation, and congestion is associated with morbidity and mortality. Haemodilution has been demonstrated a potent factor for the development of low haemoglobin levels in patients with chronic HF along with ID in multivariate analysis.^2^ Acute decompensated HF (ADHF) is linked to a poor prognosis even today, as several drugs that impact on prognosis in chronic HF are available. The therapeutic approach to ADHF has remained basically unchanged over the last decades. ADHF is characterized by increasing symptoms and signs of congestion with volume overload. However, the appropriate use of diuretics however remains challenging.^11^ Only recently, the PIONEER‐HF trial has demonstrated that early initiation sacubitril/valsartan represents a novel approach to treat stable patients with ADHF.^12^

Residual congestion at the time of discharge in acute HF or in ambulatory patients with chronic HF may identify those at high risk for adverse events.^13^ Therefore, the effect of i.v. FCM to reduce a marker for congestion, calculated plasma volume status, presented in the current issue of *ESC Heart Failure*, is definitely of great interest.^14^ In their post hoc analysis of FAIR‐HF, Onoko *et al*. confirmed that calculated plasma volume status predicts death and hospitalizations in a chronic HF cohort. In their analyses, they found that FCM is associated with significantly greater reductions in body weight and showed a trend for improvement of peripheral oedema.^14^ The observed effect fits well with the observed improvement of kidney function in patients with HF who received FCM.^7^

The relevance of this observation is underlined by the fact that diuretic resistance has a very bad prognosis in ADHF. A satisfying approach to this problem is not yet in sight. However, current randomized clinical trials such as AVANTI trial will help to shed more light on this issue in the future (ClinicalTrials.gov Identifier: NCT03901729).

Of course, further data are urgently needed for the treatment of patients with ADHF suffering from concomitant ID. The currently performed randomized Affirm‐AHF trial will provide important information on this matter (ClinicalTrials.gov Identifier: NCT02937454).

Future data on patients with HFpEF will add to the body of evidence, as the randomized FAIR‐HFpEF trial (ClinicalTrials.gov Identifier: NCT03074591).

## 5 Conclusions

Optimal fluid management is a critical issue in the treatment of stable chronic as well as acutely decompensated HF patients. The lack of a broadly used and accepted objective marker for daily clinical practice frequently makes the assessment of volume overload and euvolemia a challenge. In addition to that, diuretic resistance and the great percentage of HF patients with impaired kidney function add to the clinical dilemma. ID is frequently present and the suggested impact of i.v. iron repletion on plasma volume status together with the known positive effect on renal function makes this therapeutic approach interesting beyond the effect on energy metabolism and exercise capacity. The role of iron in acute decompensation may be even more pronounced. However, this hypothesis waits to be tested in currently running trials.

## Conflict of interest

The author has received travel grants and lecture fees from VIFOR and NOVARTIS.
